# Retraction: Historical facts of screening and diagnosing diabetes in pregnancy

**DOI:** 10.1186/1758-5996-6-59

**Published:** 2014-05-27

**Authors:** Carlos Antonio Negrato, Marilia Brito Gomes

**Affiliations:** 1 Department of Internal Medicine, Bauru’s Diabetics Association, Bauru, São Paulo 17012-433, Brazil; 2 Department of Internal Medicine, Diabetes Unit, State University Hospital of Rio de Janeiro, Rio de Janeiro, Brazil

## Retraction

This article [1] has been retracted by the Editor due to extensive overlap in text with a number of previous publications, most notably those by Mestman, Lowe *et al.* and Agarwal [2-4].
